# Outbreak of Oropouche Virus in French Guiana

**DOI:** 10.3201/eid2710.204760

**Published:** 2021-10

**Authors:** Mélanie Gaillet, Clara Pichard, Johana Restrepo, Anne Lavergne, Lucas Perez, Antoine Enfissi, Philippe Abboud, Yann Lambert, Laurence Ma, Marc Monot, Magalie Demar, Felix Djossou, Véronique Servas, Mathieu Nacher, Audrey Andrieu, Julie Prudhomme, Céline Michaud, Cyril Rousseau, Isabelle Jeanne, Jean-Bernard Duchemin, Loïc Epelboin, Dominique Rousset

**Affiliations:** Cayenne Hospital Center, Cayenne, French Guiana (M. Gaillet, C. Pichard, L. Perez, P. Abboud, Y. Lambert, M. Demar, F. Djossou, V. Servas, M. Nacher, C. Michaud, L. Epelboin);; Collectivité Territoriale de Guyane, Cayenne (J. Restrepo);; Institut Pasteur de la Guyane, Cayenne (A. Lavergne, A. Enfissi, J.-B. Duchemin, D. Rousset); Institut Pasteur, Paris, France (L. Ma, M. Monot);; Santé Publique France, Cellule Régionale Guyane, Cayenne (A. Andrieu, J. Prudhomme, C. Rousseau);; Health Regional Agency of French Guiana, Cayenne (I. Jeanne)

**Keywords:** arboviruses, Bunyaviridae, dengue-like syndrome, emergent disease, French Guiana, Latin America, Oropouche fever, Oropouche virus, Oropoucheorthobunyavirus, outbreak, vector-borne infections, viruses

## Abstract

Oropouche fever is a zoonotic dengue-like syndrome caused by Oropouche virus. In August–September 2020, dengue-like syndrome developed in 41 patients in a remote rainforest village in French Guiana. By PCR or microneutralization, 23 (82.1%) of 28 tested patients were positive for Oropouche virus, documenting its emergence in French Guiana.

French Guiana is an overseas territory of France in northern South America; 95% of the country is covered by Amazon rainforest. The remote village of Saül, deep in the rainforest, had 152 permanent inhabitants in 2017 (INSEE, https://www.insee.fr/fr/statistiques/4271842), but the actual population in 2020 was 95. The nurse of the health center keeps an updated count of inhabitants in the village, a number that was stable because of isolation during the coronavirus disease (COVID-19) pandemic. In August and September 2020, French Guiana was experiencing simultaneous COVID-19 and dengue outbreaks. Several inhabitants of Saül were treated for dengue-like symptoms, including fever and diffuse muscle pain, but rapid diagnostic testing for dengue was negative.

## The Study

**Figure 1:**
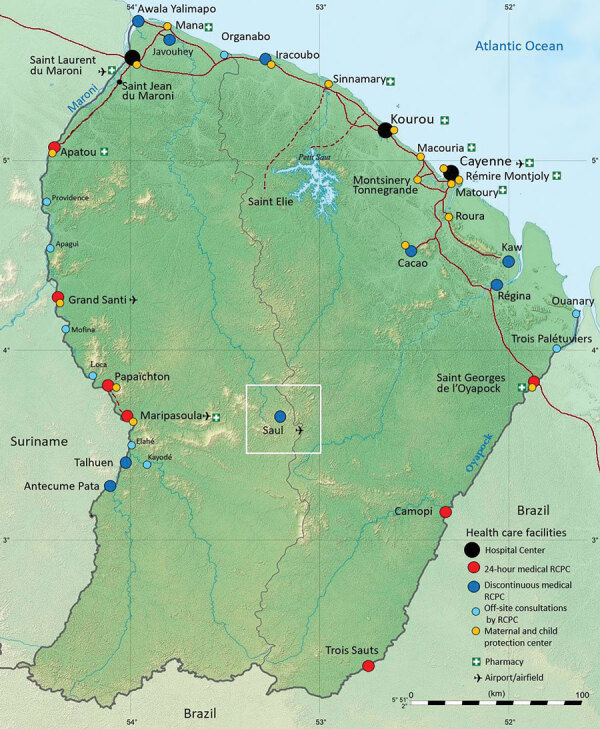
Locations of the town of Saül and 17 remote centers for prevention and care in French Guiana. Black circles: hospital centers; red circles: 24-hour remote centers for prevention and care; dark blue circles: remote centers for prevention and care (not 24-hour); light blue circles: off-site consultations with remote center for prevention and care; orange circles: maternal and child protection centers. Source: Dr. Elise Martin, Centre Hospitalier de Cayenne, French Guiana.

Saül houses 1 of 17 remote centers for prevention and care (RCPC) distributed throughout the inner territories of French Guiana (Figure 1). On August 11, 2020, a 55-year-old patient from Saül sought treatment with a dengue-like syndrome (DLS) including a marked meningeal component but tested negative for dengue. The patient was hospitalized on August 22 in Cayenne, the territorial capital. Bacteriologic, virologic, and parasitologic investigations were inconclusive. The Saül RCPC reported 15 additional patients with dengue-negative DLS during August 22–September 7. Consequently, an investigation was scheduled to begin in Saül on September 16. Sociodemographic data, clinical manifestations and evolution, and biological samples were systematically collected for each new case and, when possible, retrospectively for patients who sought treatment for DLS symptoms during August 11–September 16 (Appendix).

**Figure 2:**
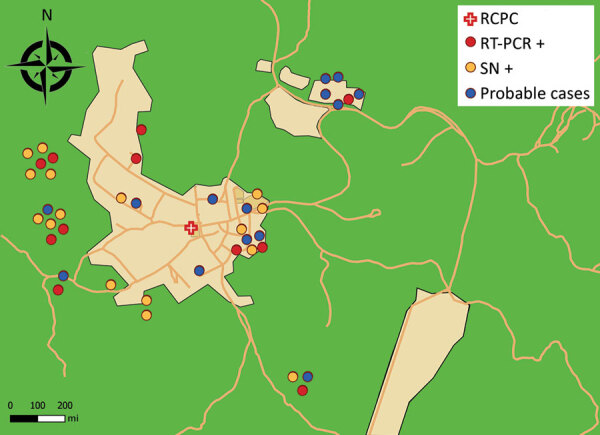
Spatial distribution of patient settlement around the town of Saül, French Guiana, and results of biologic testing for Oropouche virus by testing method. Geolocation is approximate to preserve patient anonymity. For probable cases (N = 18), samples were not taken. Green area, rainforest; light orange area, main districts of Saül; dark orange lines, forest trails. RCPC, remote centers for prevention and care; RT-PCR+, diagnosed with real-time PCR alone (N = 11); SN+, diagnosed with seroneutralization alone (N = 12).

On September 22, because results of serologic testing for common locally circulating arboviruses were negative, we performed real-time PCR for Oropouche-like virus on all available samples collected ≤5 days after the onset of symptoms (*1*). We performed viral isolations on Vero cells from PCR-positive samples and sequenced 1 isolate. Later, we performed microneutralization tests to complete biologic investigations on late serum samples. We collected clinical, biological, and anamnestic data, including localization (Figure 2), from medical and laboratory records (Appendix).

As part of the entomologic investigation, over a 48-hour period during September 30–October 2, we captured potential vectors by using 11 BG-Sentinel traps (Biogents, https://biogents.com), 5 CDC light traps (BioQuip, https://www.bioquip.com), and 1 Woodstream Mosquito Magnet trap (https://www.woodstream.com). Vector control measures, mostly aerial insecticide spraying and larval treatment, were only implemented 1 week later because of logistical constraints (lack of necessary aerial resources).

We obtained oral consent from patients to participate in the study and collected the biological samples as part of the care process. All data were collected on a standardized form and kept confidential to prevent disclosure of any personally identifiable information according to the requirements of the Commission Nationale de l’Informatique et des Libertés (https://www.cnil.fr).

During August 11–October 15, 2020, DLS was diagnosed in 41 (of 95 total) residents of Saül who sought treatment at an RCPC. Median age was 38 years (range 3–82 years, interquartile range 16–51 years) (Appendix Table 1); male-to-female ratio was 1.6:1 (Appendix Table 2). We tested blood samples from 28 patients; 23 were confirmed positive for Oropouche virus (OROV), 7 by PCR alone, 12 by microneutralization alone, and 4 by both. For the other 5 patients sampled, we were unable to confirm the diagnosis in the absence of a later sample to test for seroconversion. In addition, 17 residents, including 8 children, later reported having experienced DLS during the study period but did not visit the RPCP and therefore were not included in the study.

We obtained 5 viral isolates on Vero cells from PCR-positive serum samples; sequencing 1 of these isolates confirmed OROV infection. The attack rate in the village population was 43.2% (41/95); however, including residents with DLS symptoms who did not seek medical help would make the actual attack rate 61.1% (58/95). Few patients had underlying conditions. Symptoms by order of frequency were fever, headache, myalgia, and asthenia (Appendix Table 2). The illness followed 3 successive phases: a 2–4-day acute phase, followed by a remission phase, then a rebound of symptoms ≈7–10 days after onset. Symptom intensity decreased by the end of the second week. Persistent tiredness was reported by 73.2% patients (30/41). Elevated CRP levels of up to 10 mg/L were observed in 5 (23%) of 22 patients and lymphopenia in 10 (42%) of 24. The outbreak peaked on September 16 (Figure 2), suggesting that transmission was slowing toward the end of September. The environmental vector control intervention was first applied on September 23 and then again the week of October 6–13. The disease affected all areas of the village of Saül; the index case-patient lived on the forest edge (Appendix Figure).

In total, during 36 nighttime trapping efforts, we collected 254 mosquitoes, 242 (95%) *Culex quinquefasciatus*, and 31 *Culicoides* (biting midges), only 1 of which was *C. paraensis*, which we trapped indoors with a BG trap. We captured the other midge specimens, mostly members of the *C. guttatus* group of subgenus *Hoffmania*, near a cocoa tree orchard close to the village.

## Conclusions

Since the early 1960s, >30 OROV outbreaks have been reported, mainly in the northern states of Brazil (*2*,*3*), Peru, Ecuador (*4*), and Trinidad and Tobago, where OROV was first reported in 1955 (*5*). We report an outbreak of OROV fever in French Guiana. OROV is an arbovirus (genus *Orthobunyavirus*), transmitted through several vectors, including *C. paraensis* midges and *Cx. quinquefasciatus* mosquitoes in the urban cycle and *Aedes serratus* and *Coquillettidia venezuelensis* mosquitoes in the sylvatic cycle (*6*). Vertebrate hosts include sloths (*Bradypus tridactylus*) and monkeys (*Saguinus* spp., *Saimiri* spp., *Alouatta* spp.) (*7*). Because vectors and hosts both exist in French Guiana, the report of an OROV outbreak in this country was not unexpected.

OROV PCR is not routinely performed and serodiagnosis is not available in French Guiana; therefore, some individual cases of OROV infection not associated with an outbreak may have gone undetected. However, it is unlikely that many cases from past outbreaks went undiagnosed. Indeed, French Guiana is familiar with arbovirus outbreaks and has the resources to investigate them (*8*–*10*). Moreover, the high attack rate, homogeneous distribution of cases across the village, and different age groups affected in this outbreak imply the population had no immunity against OROV. The high attack rate could be explained by Saül’s remoteness together with factors related to the COVID-19 pandemic. The village, which is accessible only by air, has been especially affected by the COVID-19 lockdown and subsequent movement restrictions, which have isolated it even further. Also, a decrease in army presence in the surrounding forest has led to a substantial increase in illegal gold miners passing through from Brazil, which could have resulted in imported OROV. In addition, unmaintained forest trails around the village may have changed the vector density, but further entomologic studies are needed to test this hypothesis. We captured an abundance of potential vectors, especially *Cx. quinquefasciatus* mosquitoes, within the village itself. The low capture yield of local *Culicoides* spp. midges might have been linked to seasonal trends.

As described in the literature, clinical manifestations were moderately severe, and symptoms recurred among most of the patients studied (*11*). After the entomologic investigation, vector control measures were implemented in week 40. The near-exclusive presence in the village of *Cx. quinquefasciatus* mosquitoes among possible vectors suggests this species as the most plausible vector for this outbreak. However, because vectors were captured and sampled near the end of the outbreak, other potential vectors active earlier cannot be excluded. The presence of *Cx. quinquefasciatus* mosquitoes on the coast and in main cities of French Guiana and the geographic expansion of OROV in South America in recent years call for increased epidemiologic surveillance in this region (*12*).

Appendix Additional information on outbreak of Oropouche virus in French Guiana.
