# Supplementary material for: Outbreak of Oropouche Virus in French Guiana
Source: Emerg Infect Dis. 2021 Oct;27(10):2711–4. doi: 10.3201/eid2710.204760 (PMC8462337; doi:10.3201/eid2710.204760)
Supplement: Appendix — Additional information on outbreak of Oropouche virus in French Guiana. [file 20-4760-Techapp-s1.pdf]

Article DOI: https://doi.org/10.3201/eid2710.204760

Outbreak of Oropouche Virus in French Guiana

Appendix

Questionnaire

Patient n°:	Saul, the … /… / 2020	Approval: YES ☐ NO ☐
Last name:	First name:	Date of birth: _ _ / _ _ / _ _ _
Gender: M ☐ F ☐	Current job:	Phone number:

CLINICAL INFORMATION

Start Date of Signs: _ _ / _ _ / 2020

☐Fever> 38°5	☐Headache	☐Fatigue	☐Anorexia
☐Muscle pain	☐Arthralgia	☐Nausea/emesis	☐Chill
☐Cutaneous rash	☐ Retro-ocular pain	☐Diarrhea	☐Abdominal pain
Others:			

ENTOURAGE INVESTIGATION

Number of people living under the same roof: _______	Location*:
Cases in the family environment: YES ☐ NO ☐	Name:
Number of people working together: _________	Location*:
Cases in professional environment: YES ☐ NO ☐	

*Patient Mapping

WAY OF LIVING

Sleeping under a mosquito net: YES ☐ NO ☐			
Consumption of:	☐Game meat	☐Creek water	☐Others
Particular activities:			
Animals:			
____			

SAMPLING

YES ☐ NO ☐	
Date of blood sampling: … / … / 2020	A D … of the onset of symptoms

Last name: ____________________ First name: ____________________
Date of Birth: ________________ D0 (Start Date of Signs): _ _ / _ _ / 2020

D0	D1	D2	D3	D4	D5	D6	D7	D8	D9
D10	D11	D12	D13	D14	D15	D16	D17	D18	D19
D20	D21	D22	D23	D24	D25	D26	D27	D28	D29

If symptoms marked "+" to be graduated according to their intensity: light "+" / moderate "++" / intense "+++".
If no symptoms leave the box empty.
Surround the symptoms present during the different "viremic phases".

Phase 1

FEVER HEADACHE FATIGUE MUSCLE PAIN ANOREXIA
DIARRHEA NAUSEA/EMESIS ABDOMINAL PAIN CUTANEOUS RASH ARTHRALGIA
RETRO-OCULAR PAIN OTHERS, SPECIFY : ………………………………………………………….

Phase 2

FEVER HEADACHE FATIGUE MUSCLE PAIN ANOREXIA
DIARRHEA NAUSEA/EMESIS ABDOMINAL PAIN CUTANEOUS RASH ARTHRALGIA
RETRO-OCULAR PAIN OTHERS, SPECIFY : ………………………………………………………….

Phase 3

FEVER HEADACHE FATIGUE MUSCLE PAIN ANOREXIA
DIARRHEA NAUSEA/EMESIS ABDOMINAL PAIN CUTANEOUS RASH ARTHRALGIA
RETRO-OCULAR PAIN OTHERS, SPECIFY : ………………………………………………………….

Laboratory Methods

Sequencing

Viral isolations were performed on Vero cells from PCR positive samples, and 1 of the 5 isolates obtained was sequenced. Briefly RNA was extracted using Invitrogen^{TM} TRIzol^{TM} reagent according to manufacturer’s recommendations. Total RNA was reverse transcribed into cDNA using the SuperScript® III Reverse Transcriptase (Invitrogen, Life Technologies, Inc.) and random hexamers (Roche, Mannheim, Germany) under the following thermal conditions: 65°C for 5 min, 25°C for 10 min, 50°C for 1 min and 75°C for 15 min. DNA samples were fragmented by Covaris M220 Focused-Ultrasonicator (Covaris Ltd, Brighton, UK) using microTUBE-15 to 350 bp. The TruSeq Nano libraries prep kit (Illumina) was used following the instructions of the kit manufacturer except 15 cycle of amplification due to the low amount of starting materials. Sequencing was carried out on Illumina MiSeq platform at a depth of 15 million reads total. Raw

reads were processed with an in-house bioinformatics pipeline for quality and variant calling (DOI: 10.21105/joss.00352) and assembled genome using spades (PMID: 32559359).

The sequences allowed the sequencing of the 3 segments of the Oropouche virus. Raw sequences were submitted to the Sequence Read Archive (SRA): SRA accession number SRR14711849.

Microneutralization

We performed microneutralization tests to complete biological investigations in order to confirm the diagnosis of Oropouche infection on late serum samples through the demonstration of a seroconversion. Briefly, we conducted the tests in serial 2-fold dilutions of heat inactivated sera starting at 1:10 mixed in equal volume with 100 tissue culture infectious dose 50 (TCID 50) of a French Guiana Oropouche strain (obtained after isolation on Vero cells culture from a Oropouche qRT-PCR positive sample of Saul). After incubation at 37 °C for 1 h, mixtures were transferred onto 96 well tissue culture plates containing subconfluent Vero cells. The neutralization titer was expressed as the reciprocal of the highest serum dilution at which infection is blocked. A serum was considered positive for titer >20.

Appendix Table 1. Biological, clinical, and anamnestic results (continuous variables) of confirmed and probable cases of Oropouche virus infection*

Category	No.	Median (25%–75% IQR)	Min.	Max.
Test results				
Hemoglobin, g/dL	25	13.2 (12.8–14.3)	11.7	15.2
WBC, G/L	25	5.8 (4.9–8.0)	3.1	11.6
PMN, G/L	25	3.8 (2.995–4.9)	1.9	8.7
Lymphocyte, G/L	25	1.4 (0.575–2.5)	0.2	3.3
Platelet count, G/L	25	237 (197–280)	67	399
SGPT, IU/L	24	21 (14–29)	10	76
SGOT, IU/L	17	25 (21–30)	15	49
CRP, mg/L	22	4.25 (1.0–9.3)	0.2	313.7
Age, y	41	38 (16–51)	3	82
Testing delay, d†	27	4 (1.5–9.5)	0	18

*CRP, C-reactive protein; Hb, hemoglobin; IQR, interquartile range; max, maximum; min, minimum; PMN, polymorphonuclear leukocyte; SGOT, serum glutamyl oxaloacetate transferase; SGPT, serum glutamic pyruvate transferase; WBC, white blood cell
†Time between onset of clinical signs and when first biological sample was taken

Appendix Table 2. Anamnestic and clinical results (categorical variables) of confirmed and probable cases of Oropouche virus infection

	Confirmed cases			Probable cases			Total population			
Category	N	Total	%	N	Total	%	N	Total	%	P
Age, y										
<18	5	23	22	6	18	33	11	41	27	0.49
>18	18	23	78	12	18	67	30	41	73	
Sex										
Male	14	23	61	9	18	50	23	41	56	0.54
Female	9	23	39	9	18	50	18	41	44	
Confirmed cases										
Total	23	23	100	NA	NA	NA	23	41	56	NA
By test method										
PCR alone	7	23	30	NA	NA	NA	7	28	25	
PCR and microneutralization	4	23	17	NA	NA	NA	4	28	14	
Microneutralization alone	12	23	52	NA	NA	NA	12	28	43	
Medical history										
Malaria	8	23	35	4	18	22	12	41	29	0.69
Leishmaniasis	2	23	9	1	18	6	3	41	7	1
Cardiologic	3	23	13	0	18	0	3	41	7	0.20
High blood pressure	0	23	0	2	18	11	2	41	5	0.18
Evolution										
Inpatient	3	23	13	0	18	0	3	41	7	NA
Outpatient	20	23	87	18	18	100	38	41	93	
Fever										
Yes	23	23	100	16	18	89	39	41	95	NA
No	0	23	0	2	18	11	2	41	5	
Headache										
Yes	21	23	91	17	18	94	38	41	93	0.62
No	2	23	9	1	18	6	3	41	7	
Myalgia										
Yes	16	23	70	13	16	81	29	39	74	0.47
No	7	23	30	3	16	19	10	39	26	
Fatigue										
Yes	18	23	78	11	15	73	29	38	76	1
No	5	23	22	4	15	27	9	38	24	
Loss of appetite										
Yes	8	22	36	9	15	60	17	37	46	0.19
No	14	22	64	6	15	40	20	37	54	
Abdominal pain										
Yes	4	21	19	1	14	7	5	35	14	0.62
No	17	21	81	13	14	93	30	35	86	
Diarrhea										
Yes	6	21	29	2	14	14	8	35	23	0.43
No	15	21	71	12	14	86	27	35	77	
Nausea/vomiting										
Yes	7	21	33	6	16	38	13	37	35	1
No	14	21	67	10	16	63	24	37	65	
Rash										
Yes	3	22	14	4	15	27	7	37	19	0.41
No	19	22	86	11	15	73	30	37	81	
Arthralgia										
Yes	3	23	13	1	16	6	4	39	10	0.63
No	20	23	87	15	16	94	35	39	90	
Chills										
Yes	5	7	71	4	6	67	9	13	69	1
No	2	7	29	2	6	33	4	13	31	
Retro-orbital pain										
Yes	12	23	52	7	15	47	19	38	50	1
No	11	23	48	8	15	53	19	38	50	

NA, not applicable

Appendix Table 3. Biological results (categorical variables) of confirmed and probable cases of Oropouche virus infection*

	Confirmed cases			Probable cases			Total		
Category	N	Total	%	N	Total	%	N	Total	%
Hemoglobin, g/dL									
<12	0	17	0	1	7	14	1	24	4
≥12	17	17	100	6	7	86	23	24	96
White blood cells, G/L									
<4	2	17	12	1	7	14	3	24	13
≥4	15	17	88	6	7	86	21	24	88
Polymorphonuclear leukocytes, G/L									
<1.4	0	17	0	0	7	0	0	24	0
≥1.4	17	17	100	7	7	100	24	24	100
Lymphocytes, G/L									
<1	9	17	53	1	7	14	10	24	42
≥1	8	17	47	6	7	86	14	24	58
Platelet count, G/L									
<150	2	17	12	0	7	0	2	24	8
≥150	15	17	88	7	7	100	22	24	92
Serum glutamic pyruvate transferase, IU/L									
≥40	2	16	13	2	7	29	4	23	17
<40	14	16	88	5	7	71	19	23	83
Serum glutamyl oxaloacétate transferase, IU/L									
≥40	0	13	0	1	4	25	1	17	6
<40	13	13	100	3	4	75	16	17	94
C-reactive protein, mg/L									
>10	4	16	25	1	6	17	5	22	23
≤10	12	16	75	5	6	83	17	22	77
C-reactive protein, mg/L									
>50	1	16	6	0	6	0	1	22	5
≤50	15	16	94	6	6	100	21	22	95
Toxoplasma gondii IgG									
Positive	8	13	62	2	4	50	10	17	59
Negative	5	13	38	2	4	50	7	17	41
Cytomegalovirus IgG									
Positive	9	10	90	6	6	100	15	16	94
Negative	1	10	10	0	6	0	1	16	6
Epstein-Barr virus viral capsid antigen IgG									
Positive	8	8	100	6	6	100	14	14	100
Negative	0	8	0	0	6	0	0	14	0
Dengue virus or nonstructural protein 1 PCR									
Positive	0	10	0	0	4	0	0	14	0
Negative	10	10	100	4	4	100	14	14	100
Dengue, Mayaro, chikungunya, or Saint Louis encephalitis virus IgM									
Positive	0	13	0	0	3	0	0	13	0
Negative	13	13	100	3	3	100	13	13	100
Yellow fever virus IgM									
Positive/borderline	4	13	31	1	3	33	4	13	31
Negative	9	13	69	2	3	64	9	13	69
Chikungunya IgG									
Positive	1	13	8	1	3	33	1	13	8
Negative	12	13	92	2	3	67	12	13	92
Zika virus IgG									
Positive/borderline	7	13	54	2	3	67	8	13	62
Negative	6	13	46	1	3	33	5	13	38
Leptospira IgM									
Borderline	1	10	10	1	5	20	2	15	13
Negative	9	10	90	4	5	80	13	15	87
Leptospira PCR									
Positive	0	6	0	0	3	0	0	9	0
Negative	6	6	100	3	3	100	9	9	100
Coxiella burnetii IgG									
Serological scar	4	11	36	0	5	0	4	16	25
Negative	7	11	64	5	5	100	12	16	75
Malaria smear									
Positive	0	2	0	0	1	0	0	3	0
Negative	2	2	100	1	1	100	3	3	100

*G, gigagrams

Appendix Table 4. Details of mosquito species captured during the entomological mission

Trap type	Trap/nights†	*Culex quinquefasciatus*	*Cx. bonneae*	*Cx. allostigma*	Other *Culex spp.*	*Anopheles spp.*	*Wyeomyia spp.*	*Uranotaenia spp.*	Total mosquitoes (average/trap)	*Culicoides spp.*	Phlebotomine
BG Sentinel	23	206	1	1	2	0	1	0	211 (9.2)	1*	1
CDC light traps	11	10	0	0	2	2	0	1	15 (1.4)	30	273
Mosquito Magnet	1	27	1	0	0	0	0	0	28 (28)	0	0
Total	35	243	2	1	4	2	1	1	254	31	274

**C. paraensis*
†Trap/nights, no. traps × no. nights

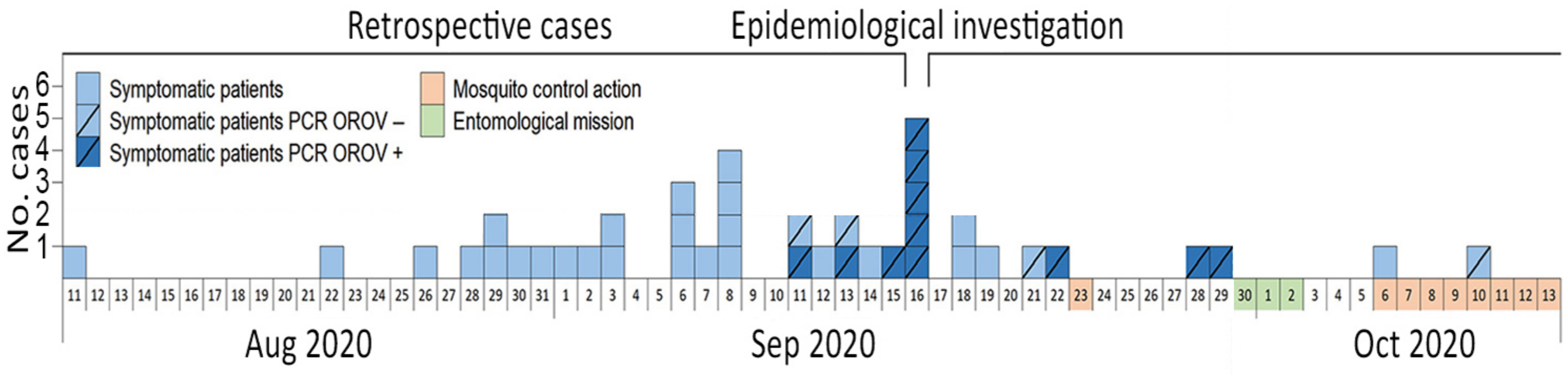

Appendix Figure. Patient settlement spatial distribution and OROV biological results. Geolocation is deliberately approximate to preserve anonymity. RCPC: remote centers for prevention and care; RT-PCR+: patients diagnosed by real-time PCR alone (N = 11); SN+: patients diagnosed by seroneutralization alone (N = 12); not sampled: probable case (N = 18); green: rainforest; light orange color: down town; dark orange lines: forest trails.
